# Unveiling the Evolution of Eldercare Facilities in Rural China: Tracing the Trajectory from Eldercare Support Pattern and Service to Facilities for the Aging Population

**DOI:** 10.3390/healthcare11182474

**Published:** 2023-09-06

**Authors:** Ziqi Zhang, Zhu Wang, Zhi Qiu

**Affiliations:** 1 School of Design, Shanghai Jiao Tong University, Shanghai 200240, China; 2 Institute of Architectural Design and Theoretical Research, Zhejiang University, Hangzhou 310058, China

**Keywords:** eldercare facilities, eldercare support patterns, eldercare and healthcare services, rural China, political framework

## Abstract

The phenomenon of population aging in rural China presents a compelling societal challenge, necessitating a growing demand for both the quantity and quality of facilities supporting the needs of older people. However, a lack of comprehensive understanding concerning the underlying mechanisms that drive the emergence of these facilities, coupled with the distinctive historical backdrop and social development stage of the nation, engenders complexities in achieving sustainable rural eldercare services. This paper endeavors to comprehensively elucidate diverse eldercare facility types in rural China, delineating their intrinsic characteristics and prerequisites for construction. Additionally, the research delves into the political and economic contexts and advancements in healthcare and eldercare services, culminating in the formulation of an integrated framework that interconnects eldercare support patterns with the political landscape and public service provisions. The implications derived from this nuanced framework provide insightful reflections on significant historical transitions, intricacies faced by rural eldercare facilities, and strategic pathways for fostering future eldercare service delivery systems and allied facilities. The paper’s findings furnish insights for bolstering the well-being of the aging population in rural China and lay a substantive foundation for addressing the evolving requisites of eldercare within this distinctive context.

## 1. Introduction

Population aging has become one of the most significant societal developments in China. China’s older population is anticipated to reach 300 million by 2025, constituting 21.4% of the overall population. This figure is expected to further increase to 400 million by 2050 [1]. In recent decades, there has been a widening demographic gap in terms of aging between economically developed urban areas and rural areas [2]. In rural areas in 2020, the percentage of senior people aged 60 and older and 65 and older was 23.8% and 17.7%, respectively, 8.0% and 6.6% more than in urban areas [3].

The aging issue presents a greater challenge in rural regions of China. The large-scale youth migration from rural to urban areas is tied to the rapidly increasing rural aging rate [2,4], which is evidenced by the notable decline in rural population, coupled with a slight increase in the proportion of older rural residents [3]. This transition leads to the breakdown of familial caregiving structures and a subsequent rise in the rural dependency ratio. The number of empty nest families is also on the rise: it is projected that by 2050, the proportion of older people left behind in rural areas will reach 26.1%, approximately three times that of urban areas [3]. Nevertheless, the rural older population exhibits a 16.3% higher likelihood of depending on family members (47.7%) as compared to their urban counterparts [1]. It is foreseeable that there will be a sustained increase in both the quantity and quality of demand for community services and eldercare facilities in rural areas. As discussed, the decline in older villagers’ personal ability to care for themselves and the reduction in the size of conventional family structures have led to a number of unavoidable external changes, making the delivery of eldercare necessitate SOAC, a robust and comprehensive model that addresses the needs of older individuals by considering societal input and social forces as a foundation for providing services [4]. It is also the result of a gradual shift in the attitudes of older villagers towards institutional arrangements as a result of their acceptance of changing realities, comprehension of various external measures that facilitate the expansion of the eldercare industry, and access to increasingly advanced economic and informational resources.

However, notwithstanding the recent enhancements in the accessibility of communal healthcare and eldercare services, rural communities continue to grapple with disparities. On the one hand, China’s existing pension system exhibits uneven coverage due to the persistent urban–rural dual economic structure. The New Old Age Insurance System for Rural Residents is inadequate in meeting the fundamental living requirements of the older population [1,4]. On the other hand, the supply and quality of eldercare services provided to older villagers remain comparatively inadequate. Numerous rural eldercare facilities currently in existence cannot be compared to nursing homes or even to the notion of assisted living facilities used in Western culture [5]. In general, older people in rural areas are more likely to have poor health or be unable to care for themselves than their urban counterparts. The absence of effective health risk management or appropriate service provision may exacerbate a self-perpetuating cycle of poverty and unfavorable living conditions [6]. Overall, older persons in rural regions are more prone to poor health and self-care challenges compared to their urban counterparts in a context where family-based informal care is diminishing, and external resources, including financial support and public goods for eldercare or health services, are yet to significantly bridge the gap.

To tackle the challenges associated with aging in rural China, it is imperative to gain insights on how to capitalize on policy opportunities, adapt to the constantly changing eldercare ideologies, acknowledge the challenges posed by the rapidly evolving rural system, and develop customized policies that cater to the diverse needs of older people. Previous literature mainly discussed the changes in medical and health financing regarding rights and interests under the Rural Cooperative Medical Insurance System [7,8,9,10,11,12,13], pension security and healthcare services [14,15,16], the impact of social structure changes on the way of supporting the aged [17,18], as well as the accessibility of medical facilities and the spatial inequality between urban and rural areas [19]. These studies cover the eldercare system in rural China from the aspects of economy, policy, or planning from macro perspectives. In limited research on institutional care, most studies have focused on seniors’ attitudes toward institutional care and their influencing factors [20,21,22,23,24], or on long-term care in urban settings and corresponding community-based agencies [25,26]. Only a small number of studies have focused on the current status and development of eldercare amenities in rural China [27] or the quality of life of older people residing in these facilities [28,29]. Research focusing on the daily life, needs, and behaviors of rural ‘left behind’ senior groups, as well as comprehensive investigation on the impact of the built environment or care services on their quality of life [30], has only begun to develop in the past decade [31]. The discourse and incorporation of indigenous knowledge from various rural eldercare facilities into a knowledge framework are severely hampered by the lack of a comprehensive picture of rural eldercare establishments in China.

China is confronted with significant challenges, primarily centered on the development of an eldercare service delivery model that effectively integrates the country’s distinct social, cultural, economic, and political attributes [32]. Architecture acts as one of the referents to understanding broader social, political, and historical contexts [33,34]. As Lefebvre [35] notes, only by understanding the logic of the production of space can we control, utilize, and create space. In order to better understand the eldercare facilities in the midst of rural development and how they attach or respond to the system, this paper will unpack the evolutionary logic and issues of rural eldercare facilities from a macro perspective of political and economic historical change. This will help to better understand the potential types, nature, ways of design, and operations of rural eldercare facilities in the current and forthcoming milieu. Specifically, the paper first summarizes the fundamental characteristics, construction prerequisites, and developmental trajectory of various categories of eldercare facilities in rural China, drawing upon field-paper reports. Secondly, by exhaustively curating and analyzing policy documents and literature relevant to the historical context since 1949, the paper systematically unfolds the intricate political and economic background of rural China, along with the transformative dynamics of healthcare and eldercare services. It culminates in the construction of a comprehensive framework that facilitates the understanding of the operational mechanisms governing eldercare facilities. With this methodological apparatus in place, the paper systematically tackles three primary dimensions: (1) unraveling the interplay between the evolution of eldercare facilities and significant historical junctures that have shaped them; (2) summarizing the prevailing challenges encountered by each kind of rural eldercare facility from a more intrinsic perspective; (3) proposing forward-thinking for prospective eldercare service delivery systems and the concomitant infrastructure.

## 2. Eldercare Facilities in Rural China

In China, the provision of institutional care for the older adults is primarily facilitated through both social welfare and healthcare systems. The healthcare system encompasses rehabilitation units within geriatric hospitals, general hospitals, and psychiatric facilities, while the social welfare system comprises assisted living facilities and senior apartments [27]. The fact that they are continually changing in response to technological improvements and adjustments in rural macro policies adds to their complexity. Currently, rural China primarily has the following types of eldercare facilities.

- Rural nursing home/‘Wubao’ service institute. Nursing homes, the earliest form of eldercare service facilities, were established in rural China in 1956 and developed based on the ‘Wubao’ system (‘guarantees’ of providing five kinds of fundamental necessities including clothing, food, shelter, transportation, medical care, and end-of-life arrangements for older persons without labor ability, income, or children) during the period of the collective economy [30,36]. Policy advocacy during the era of the People’s Commune led to nursing homes seeing remarkable growth; by 1959, 150,000 such facilities across the nation had taken in over 3 million older individuals who were widowed or alone. Due to inadequate management after three years of natural disasters and the Cultural Revolution, many nursing facilities were repurposed for agricultural production or ‘Huazifang’ (rooms occupied by beggars) and did not begin to recover until the Household Contract Responsibility System was launched. A significant step in the standardization and formalization of nursing homes, in line with the ‘Wubao’ system, was taken in 1997 when the *Interim Measures for the Management of Rural Nursing Homes* established their clients, economic source, management, and operational structure as a rural collective welfare institution. The *Decision of the Central Committee of the Communist Party of China and the State Council on Strengthening the Aging Work (*2008*)* pushed conditional nursing facilities to open to all seniors but still prioritized ‘Wubao’ seniors. The nursing home’s dependence on subsidies from the government or collective is intricately linked to its target demographic and characteristics, making it especially susceptible to changes in the political environment; they have experienced a cyclical trend of growth, decline, stabilization, and institutionalization, which has been shaped by policy interventions during the People’s Commune, economic challenges arising from prolonged natural disasters, and the adoption of the ‘Wubao’ system by township governments operating under the Household Contract Responsibility System.
- Rural ‘Starlight’ senior center. China’s Ministry of Civil Affairs launched the *Community Senior Welfare Services ‘Starlight’ Program* in 2001 on a national scale with the aid of 20% of the welfare lottery revenue as financial assistance. From 2001 to 2004, a total of CNY 13.4 billion was invested in building 32,000 ‘Starlight’ senior centers. It was believed at the time that more than 30 million seniors would benefit from the services of recreational activities, home visits, first aid, nursing intervention, or medical care provided by the centers [32,37]. Zhejiang Province stood out as one of the few regions that offer specific construction guidelines for ‘Starlight’ senior centers situated in rural areas, as opposed to the majority that adhered to the construction norms that are commonly applied in urban contexts. Currently, following the extensive implementation of home-based care, the majority of rural ‘Starlight’ centers have been repurposed as daycare institutes, while some have not undergone renovations and have been plagued by unsustainable financial input, being occupied by private or misappropriated use.
- Mutual support ‘Happiness Home’ (MSHH). In 2008, a facility for mutual aid among older villagers was initiated in Qiantun Village located in Hebei Province. It is a self-managed and mutual aid facility for the older villagers in which inhabitants share the cost of public utilities and daily living expenses. It was officially promoted by the government in 2011 with the name ‘Happiness Home’ since it embodies the ‘Antuzhongqian’ tradition (settling down in the place in which one has long lived) and local collective and government economic realities. In 2013, the Ministry of Finance and the Ministry of Civil Affairs issued the *Project Management Measures for the Central Special Lottery Public Welfare Fund to Support Rural ‘Happiness Homes’,* backing the construction of rural MSHHs with national finance. As of 2014, a total of 79,521 MSHHs have been established in rural China [38]. Currently, these facilities may not necessarily be named ‘Happiness Homes’; they are called elderly mutual aid centers in Guizhou or are part of ‘Mulindian’ (neighborhood points) in Shanghai.
- Rural home-based daycare service center. In 2008, the National Office for Aging of the Ministry of Civil Affairs issued the *Opinions on Comprehensively Promoting Home-based Eldercare Services*, which mandated the establishment of a comprehensive senior welfare service facility that integrates institutional care, home-based care, and community care in rural communities by leveraging existing facilities and resources. This is one of the main types of eldercare facilities promoted in rural China after communityization. The national standards for home-based daycare service centers largely apply to urban communities rather than villages when it comes to construction requirements. A few regions, like Zhejiang, have established village-level guidelines for these facilities. It was not until 2023 that rural home-based care service facilities had their first nationwide design standard.
- Rural clinic/health service center. The first National Health Work Conference in 1950 proposed the establishment of collective-owned joint clinics in rural areas, as well as the establishment of health centers in counties, districts, and administrative villages, forming a three-level network of healthcare services. In the agricultural cooperation period, various regions successively launched the construction of farmer-funded and collectively owned health stations, as well as health and medical stations established by agricultural cooperatives. During the period of the People’s Commune, those institutions and manpower were integrated into the commune’s healthcare system. The health centers in townships were integrated into the health stations managed by production brigades, and to facilitate farmers’ access to medical care, each production team had a clinic. Later, the three-level organization composed of commune hospitals, health stations of the production brigade, and clinics of the production team was determined by the *Amendment to the* Regulations on *the Work of Rural People’s Commune*, *Draft Opinions on Adjusting Rural Basic Health Organization* and other documents, with the principles of decentralized, small, and multi-point settings proposed. Rural clinics were given their roles of preventive care, primary diagnosis, and treatment of common injuries and diseases within the rural healthcare service network. Since the construction of rural communities began, most of the clinics have been renamed as health service centers.

**Figure 1:**
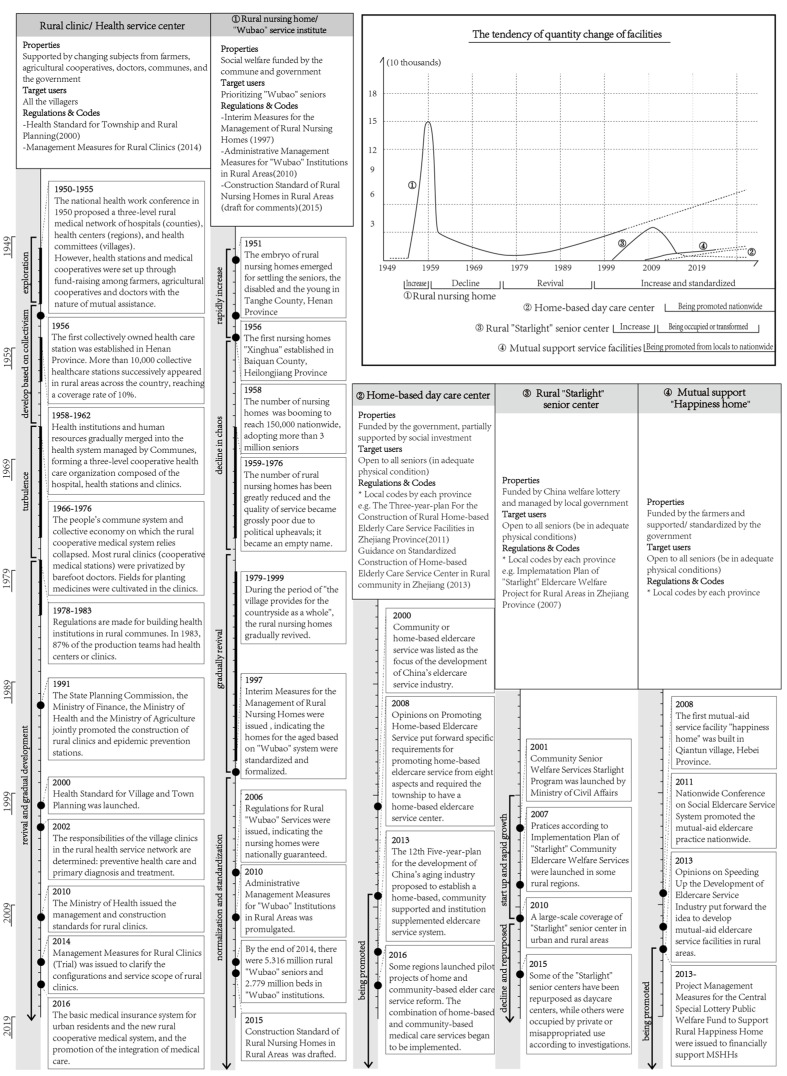
The chronology of eldercare facilities in rural China since 1949.

The graphical representation in Figure 1 provides an overview of the fundamental characteristics, significant historical milestones, and evolution of the primary types of healthcare and eldercare facilities in rural regions from 1949 to the present day. The timeline format illustrates the main alterations in the quantity and attributes of these facilities over time.

## 3. Interpreting the Evolution of Eldercare Services in Rural China

### 3.1. Evolution of Eldercare and Public Service Support Pattern Services in Rural China

The evolution of social security in rural areas has followed a distinct trajectory from that observed in urban areas, in accordance with the socioeconomic development of these regions. In ancient Chinese society, external aging support was seldom mentioned as the prevailing emphasis on family and filial piety placed responsibility for the care of older members within the family unit [39]. In the modernization process, the economic and social transformations in rural regions have led to a decline in the cohesion and backing provided by intergenerational familial care [40], which gradually resulted in a significant dearth of external assistance for older individuals. The implementation of the socialist collective economic system facilitated the consolidation of the ‘Wubao’ system which was further reinforced by the collective authority. However, most older people still relied on family/clan support and self-care. The collective system started to decay around 1978 when the People’s Communes left the historical stage. In 1992, the *Basic Scheme of Rural Social Pension Insurance at the County Level* was released, trying out a *personal saving* insurance system. A decade after, the *New Rural Social Old-age Insurance* in which ‘the premiums are paid mainly by the personal savings account, supplemented by collectively pooled subsidies and supported by government policies’ began to be implemented. Although the new system was still unstable in terms of security level and in service quality [41], it achieved a wide coverage of 95.7% of the population in 2011, reducing inequality between rural and urban areas [8,16]. In general, the evolution of the eldercare system in rural China primarily entails enlarging the eligible demographic and augmenting the available resources to counterbalance the gradual decline of familial care as the nation’s economic and social welfare levels advance. On the one hand, the rural pension security system has developed from its roots in the ‘Wubao’ system to the New Rural Social Pension Insurance, which benefits a wider range of rural residents. On the other hand, the allocation of rural land ownership and management rights between collective and private units subsequent to land reform results in the fragmentation of the obligation for eldercare, thereby fostering collaboration and mutual support among society, communities, families, and individuals in the provision of resources for the aging population [23,42], thus increasing the choice of eldercare support patterns.

Throughout history, rural public services, encompassing vital elements of public eldercare and healthcare services intricately linked to the aging population, have also experienced significant and transformative transitions. In the early 1950s, farmers could only obtain a modest amount of mutual aid. Throughout the collectivization process’s developing phase, the highly centralized rights and resources from the People’s Commune sprouted and nurtured a limited public eldercare service that primarily served the ‘Wubao’ households. However, the existence of the People’s Commune continuously aggravated the inequality between urban and rural areas in terms of household registration, employment, social welfare, medical care, and healthcare, resulting in a dual structure that prevented service resources flowing from urban to rural areas. After the implementation of the Household Contract Responsibility System in 1978, the power of the People’s Commune declined, failing to further provide eldercare service in rural areas. In 1982 when the villagers’ committee system was established, the social support for the elderly expanded to a wider coverage of the majority. However, the characteristics of ‘separation of urban and rural areas’ and ‘co-existence of village and commune’ still hindered the development of rural public service provision and circulation systems. Formal eldercare services in rural areas were still in serious shortage. The Village Renovation and Rural Community Transformation was launched in 2006 with the goal of establishing community-based rural public services and equalizing access to urban and rural public services, which put the provision of public eldercare services on the right track. Following 2008, there was a promotion of broadening the scope of socialization to encompass community-based care and optimizing the utilization of home, community, and institutional services, which was crucial in addressing the escalating demands of older individuals residing in rural regions.

At the same time, the public healthcare service in rural areas has also experienced a series of changes. In 1950, health stations, medical stations, and other health institutions were established by raising funds from agricultural cooperatives and villagers. After the founding of the People’s Commune in 1958, these institutions and human resources were merged into the healthcare force of the Commune, and the three-level healthcare service system supported by the commune and subsidized by the government was gradually established and improved. Although this system was unstable during the declining period of the commune, the medical problems of rural residents could be solved by a large number of ‘barefoot doctors’ (informal healthcare providers with basic medical training in rural areas) and homemade medicines. The ‘barefoot’ doctors promoted the Cooperative Medical System (CMS), which attained its zenith in the mid-1970s, ensuring medical coverage for approximately 90% of the rural populace and fundamentally altering the landscape of healthcare disparity during the planned economy period [13]. However, the CMS existed only in name after the economic reform and the collapse of the People’s Commune with the coverage reduced to 4.8% in the country in 1989 [43]. In the early 1990s, the promulgation of *Opinions on the Establishment of a New Rural Cooperative Medical System (NCMS)* was released, and the NCMS, which was based mainly on a financial pool of a serious-disease scheme, was finally launched in 2003 [11,19]. This system tried to promote the overall planning and allocation of rural cooperative medical care and established a rural social health service network with public ownership as the leading factor and multi-ownership as the company. In 2016, *Opinions on Coordinating the Basic Medical Insurance System for Urban and Rural Residents* put forward the requirements and principles for coordinating the basic healthcare insurance system for urban and rural residents.

Drawing upon a perspective widely shared among scholars, which asserts that the development of eldercare support patterns can be interpreted through the lenses of economic security, developmental stage, and political orientation [44,45,46], we categorize the progression of rural eldercare policies since 1949 into four stages. Meanwhile, public eldercare and healthcare services have traversed a trajectory from self-organized and self-financed mutual assistance among villagers to the gradual integration of social welfare supported by Collectivism. They also experienced a tumultuous period following the weakening of the commune system to the new era of public service supply after the revolution of the basic autonomous organization (village committee and rural community). The development of public services in rural China can be divided into four stages according to the administrative system and the influence of urban–rural structure. Similarly, the trajectory of public healthcare services can be demarcated into five stages (Table 1).

### 3.2. A Framework from Support Patterns and Service Delivery to the Eldercare Facilities in Rural China

The eldercare support pattern, combining the above analysis of its evolution, can be understood within the historical context, influenced by economic conditions, political ideologies, and social structures by involving two key processes: the ‘supply’ and ‘distribution’ of resources. On the one hand, the ‘supply’ process is contingent upon productivity and the social security system. The former sets the theoretical upper boundaries for resource provision, highlighting the challenge of ‘growing old before getting rich’ confronting China. The social security system and the culture (including traditions, ethics, the common family structure, etc.) together determine the content and quantity of the actual supply of formal and informal resources. It is worth noting, among other things, that in the supply process, public services are particularly important in rural areas, given the social context of low productivity and economic conditions, as well as the ‘inherent vulnerability’ [47] of agricultural workers. However, these services are either impractical for the market to provide or economically unviable, emphasizing the importance of government intervention and public policies in ensuring service provision. The concept of externalities in welfare economics further emphasizes government intervention and policy support to attain efficiency in resource allocation for public goods [48]. At the same time, many Chinese scholars advocate for a diversified welfare responsibility [49], as outlined in the welfare triangle or welfare pluralism, aiming to address the unique challenges posed by market and government failures under China’s distinct pension systems and social structures. Historical developments reveal this process and trend as there was a period of insufficient supply of rural eldercare services until the social security system built up from nothing (when people had to completely depend on self or family support) and transitioned from sole reliance on the government to the involvement of multiple stakeholders. On the other hand, a pension serves as a means of redistributing income through social security systems [50], thus making the process of ‘distribution’ primarily reliant on political settings and the management of resources. In addition to government-provided security, variations in traditions, ethics, values, and other sociocultural factors that evolve with social development can influence the availability of informal support for older individuals. For instance, the traditional expectation of family members cohabitating and providing resources, respect, and care to their older relatives has shifted to a scenario characterized by ‘filial piety at a distance’ and a growing number of empty nesters due to the migration of children for employment opportunities during industrialization [51]. Thus, the ‘actual distribution’ available to the older villagers is determined by a combination of nationwide public goods (such as economic support, pensions, and formal services) under the prevailing political system, as well as cultural traditions, including ideological attitudes and filial piety support patterns, which could vary across regions and further impact the distribution of eldercare resources.

The provision of rural public services is considered to have exhibited three kinds of ‘fragmentation’ [52] in its history: system isolation, resource scarcity, and government competition, which, like the pattern of eldercare support, also align with specific historical contexts characterized by economic, social, and political factors. The same research also suggests that to address this fragmentation, a comprehensive governance perspective is necessary, involving policy and institutional arrangements at the macro level, resource integration and organized supply at the meso level, and expression of demand and supervision by villagers at the micro level [53]. By establishing an interactive and coordinated system that encompasses mechanisms, resources, and actors, the aim is to mitigate the negative effects of fragmentation on the provision of rural public services. Within the framework established in this paper, public eldercare services are conceptualized as a process whereby the content (and quantities) of services from various suppliers is directed through distribution channels (utilizing different administrative structures) to meet the specific needs of villagers. This aspect can be understood as the implementation of the aging policy and the operationalization of the eldercare support pattern. One crucial component in the efficient delivery of public services is the circulation channel for resources, of which rural grassroots organizations are fundamental. These organizations play a vital role in not only bringing resources into rural areas but also effectively distributing them. China’s rural grassroots organizations have historically served as a vital ‘connecting link’ in mediating the relationship between the nation and rural community members by assisting authorities in accomplishing tasks, mobilizing villagers for collective actions, voicing the preferences of farmers regarding public goods, and representing farmers’ preferences regarding public goods [54]. For example, the People’s Commune, as a rural grassroots organization, has played a significant role in ensuring the stability of rural society and the provision of public services over an extended period. Furthermore, addressing the needs of villagers/users has become increasingly important. Village-level organizations, with their allocation of resources for public goods, can employ ‘distributive democracy’ [54] through representatives’ meetings to effectively voice the preferences of the majority of villagers, thereby better serving specific groups.

**Figure 2:**
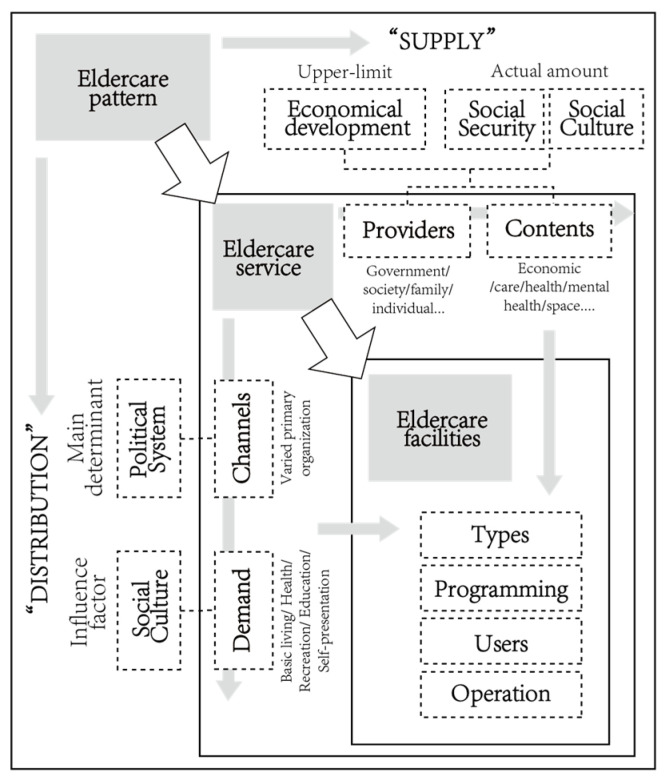
The framework from the eldercare support pattern, to public services, to the corresponding facilities.

In Western societies, the ‘structuration’, a process that involves the conversion of capital into the physical urban form, is shaped by the dialectical interaction between the nation and civil society, with civil society wielding significant power. In contrast, the Chinese context involves the active participation of all levels of government in shaping the built environment; its spatial production is characterized by a collectivized approach, where the (local versus central) government plays a central role [55]. Lefebvre’s theory of the production of space and the spatial triad offers a significant framework for comprehending the evolutionary process of constructed space and its social structure [56]. He argued that space is not only a physical outcome resulting from specific social relations but also a reflection of the underlying abstract relationships. It emerges through the interaction of political and economic dynamics within a given society. He emphasized that ‘absolute space’ cannot exist without its own inherent logic. This perspective links design to a dynamic and political understanding of space. Therefore, China’s unique sociocultural, economic, technological, and policy factors contribute to its distinct spatial production parameter [57,58], which can be employed to elucidate the development of rural eldercare facilities within a broader institutional and cultural context. They represent an entity that encompasses the content and extent of services, bridging the political and economic realities of rural areas throughout different historical periods. By tracing the changing patterns of elder care support and public services in rural China, we elucidate their structure and nature, linking the institutional and cultural contexts to the final architectural type of ‘outcome’ and developing a framework. This framework demonstrates the correspondence among the eldercare support pattern, eldercare/healthcare services, and eldercare facilities. The eldercare support pattern is the supply and distribution framework of the resources that could be used for eldercare based on the macro background; the public eldercare/healthcare service reflects the specific process of supply and distribution, outlining how resources flow from providers to recipients. Eldercare facilities are the final outlet and observable ’phenomenon’ that separates or integrates the needs of older users and carries them, based on the available resources and the designated roles within the political organization of the village and the integrated system (Figure 2).

## 4. Discussion: The Past, Present, and Future of Eldercare Facilities in Rural China

**Figure 3:**
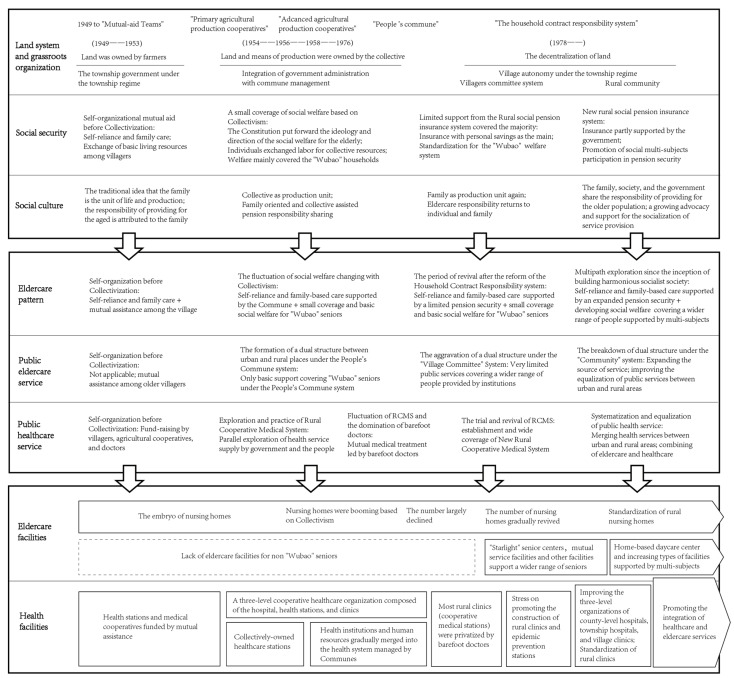
The evolutionary track of support pattern and service delivery to the types of facilities.

The primary purpose of this framework is to review the progress made in the establishment of eldercare facilities in rural areas. This framework allows us to sort out the coupling between changes in eldercare facilities and the key historical points, combining the political and economic context, land policies and autonomous organizations, economic development, security system, and social culture of a specific period with changes in eldercare support patterns, stages of development of public eldercare and healthcare services, and, most importantly, eldercare facilities. The interpretation of the connotation and connection between ‘eldercare support pattern–service–facilities’ may help to explain the fundamental reasons and logic of the production and evolution of eldercare facilities in rural China from a systematic theoretical perspective (Figure 3).

The second purpose of this framework is to critically re-examine the current problems in eldercare facilities in rural China. Rural eldercare facilities still have subpar conditions and a low utilization rate after a lengthy period of construction and development. This phenomenon’s underlying problems might lie in the following aspects according to the framework.

- The standardization of eldercare facilities surpasses the current economic development. Existing construction standards for rural eldercare facilities exhibit a lack of clarity regarding functional positioning, with a tendency towards incomplete functional settings and high construction requirements. Specifically, the construction standards for rural nursing homes catering solely to the ‘Wubao’ seniors even surpassed those for more metropolitan communities, plainly exceeding the capacity dictated by the level of rural development. As a result, most rural nursing homes were inclined to keep the activities that call for less space and services, such as recreational and outdoor activities, and reduced difficult-to-implement functions that require more resources, both material and human, like rehabilitation. This issue may also be attributed to the absence of clear positioning for each eldercare facility, including its role in providing public services in conjunction with other public facilities and its interaction with the rural public space system. As a result, despite having different categories, the constructed eldercare facilities lack diversity. The disparity and disorder arising from high construction standards but substandard construction, coupled with high service demands but prevailing unaffordability, led to a surplus of 475,000 unoccupied beds in rural nursing homes, with a utilization rate of only 78%.
- The availability of resources for the older population residing in rural areas remains inadequate, particularly concerning healthcare services. Several research studies have highlighted the insufficient benefits of primary healthcare services in rural communities. This is evident in the scarcity of standard medical diagnostic equipment, such as blood or urine tests, in specific township health centers, as well as the fact that unlicensed healthcare personnel is prevalent, with a rate exceeding 30% in urban health centers and 75% in rural clinics [59]. In addition, eldercare facilities and amenities suffer from a lack of continuous funding. This is especially problematic for the ‘Starlight’ senior centers, which depend on welfare lottery funding. The lottery monies were used as a transitory, one-time investment without market-based operational measures to improve fund utilization; thus, such facilities were susceptible to disorder and deterioration.
- The current political system presents a hindrance to the efficient distribution of resources. A critical concern that requires attention in rural senior facilities is the sustainability of operations post-construction. It is important to recognize that while there is a continuous demand for financial input, relying solely on government assistance is not feasible, especially in the rural context. Locating the facility’s identity in the administrative and resource provision system is the first and essential step in activating its own operational capacity, which can help rationalize the distribution of the object demands to be satisfied in accordance with the resources available to the various eldercare facilities in a system. This approach mitigates the inefficient replication of building and subsequent functioning, while also addressing the potential hindrances to capital investment arising from the institution’s equivocal character. For example, the regulatory framework pertaining to rural nursing homes lacks clarity, as observed in the Regulations on Rural ‘Wubao’ Services. The aforementioned regulations assign management responsibilities for the nursing homes to both the Civil Affairs Bureau and the township government. The lack of clarity in the management function has led to functional misalignment, an unclear division of authority and responsibility, and increased burden on townships in managing rural nursing homes. Moreover, it renders rural nursing homes incapable of accepting social donations as independent institutions.
- The passive shift of responsibility for eldercare services. The dearth of formal services in eldercare facilities has necessitated compensation from informal, non-professional services. For instance, the eldercare services promised in the norms of nursing homes, the ‘Starlight’ senior centers, and home-based senior care service centers are severely lacking in actual operation, whereas happiness homes have shifted the majority of senior care responsibilities back to the older villagers themselves from the start. Consequently, older residents had to rely on self-assistance and mutual aid to address the gaps in service provision. Furthermore, the absence of a specified target population in the requirements guiding eldercare facilities, coupled with the passive and autonomous nature of their operations, has resulted in a dearth of professionalism in the services provided. This has led to a lack of acceptance of older individuals most in need of assistance, including those with diverse physical conditions, those requiring assistance with self-care, as well as those with disabilities and paralysis.
- The inhibition of traditional cultural concepts regarding institutionalization. The evolution of rural eldercare facilities reveals that they have traditionally been associated with nursing homes for the ‘Wubao’ seniors, a perception that is commonly linked to destitution and abandonment in the minds of rural inhabitants. Rural seniors show a lower acceptance of external assistance compared to their urban counterparts, being less inclined towards relying on institutional care or other care alternatives. Furthermore, they exhibit a reluctance to express their ‘real’ demands, thereby posing challenges to a demand-based provision of eldercare services and facilities.
- A discrepancy exists between the supply of eldercare services in rural areas and the corresponding demands. The seemingly contradictory status quo of vacancy and absence actually reflects the lack of awareness of the rural situation and the needs of the older villagers during the pre-construction period. The absence of appropriate construction standards has led to the majority of construction specifications for rural eldercare facilities being mere replicas of urban specifications. This approach failed to cater to the unique requirements of rural seniors and the distinctive features of the rural environment, resulting in a mismatch between the available resources and the actual needs [60].

Referring to the issues above, this framework’s third purpose is to offer recommendations for upcoming eldercare facilities in rural areas. Currently, China’s pension policy is predicated upon a multi-level eldercare service system that relies on the collaborative efforts of households, communities, and institutions. The present scenario poses a crucial inquiry regarding the development of an eldercare framework that can effectively conform to contemporary social, economic, and political ideologies. This framework must also optimally utilize all accessible internal and external resources while catering to the requirements of older individuals residing in rural regions. ‘Homecare as the basis’ emphasizes the idea of homes as both living spaces and the primary source of eldercare for families. However, the unabated trend of the core family’s shrinking and the deterioration of family support makes it necessary to remove some of the caregiving duties from the family by enhancing community support and enlisting the help of older adults to lessen the physical and emotional dependence on family support. Secondly, ‘community as the support’ suggests the administrative responsibility of the community as the current organization management system in rural areas, which will be, as this paper points out, responsible for the reception of external input resources from the upper level and the unified integration of internal resources, so as to form a formal support network. The sociological connotation of the community also indicates the possibility of forming a mutual aid or informal support network among the residents. Thirdly, ‘institutions as the supplement’ indicates the institution should be the place where community services are provided. The service includes both the institutional services for older adults and the transfer station for the service output to the family. Therefore, the current eldercare support pattern of ‘home-based, community-supported, and institution-supplemented’ requires the partial separation of eldercare responsibility from the family by external support that comes from the community and institution. That is, to receive external resources and integrate internal resources by taking the rural community as the unit and the institutes in the community as the intermediary. The distinctiveness of rural regions is evidenced by the segregation of various functions from families, the heightened efficiency of social support utilization necessitated by economic constraints, and the disparate ratio resulting from the amalgamation of formal and informal support.

The implication for policymaking may be mainly from the binary processes of supply and distribution and the four aspects of the economy, social security/culture, political system, and social culture. Potential policy orientations encompass various aspects to enhance rural economic development, long-term care for older individuals, social security, rural land use, family responsibility in caregiving, mutual aid, and informal care, promoting active aging and social participation among the aging population. These directions may include the implementation of supportive policies for industrial development and urban–rural cooperation, construction of facilities catering to the needs of older individuals, interconnecting social security between urban and rural areas, modifying rural land use policies, encouraging shared family and community responsibility for eldercare, guiding and standardizing mutual aid and informal caregiving, and advocating active aging and increased social engagement for the older people. In terms of eldercare service delivery, most importantly, it is necessary to determine the contents of eldercare services according to the features of the rural aging group through qualitative and quantitative methods, as well as to promote the institutionalization and standardization of relevant evaluation of demand and user segmentation. The integration of urban and rural eldercare and healthcare resources and the separation of responsibilities of each relevant subject in the formal and informal service network should be clarified. Concurrently, an input, circulation, and feedback process for eldercare services, founded on the administrative management system, should be established as the core of public service organizations to cater to their needs, thus forging a cohesive rural community public service network. In the aspect of community planning, the rational and optimized positioning and functionality of current public facilities are essential. Scholars have advanced theoretical models of rural community service provision, advocating support for the naturally occurring system of mutual assistance in rural areas, reinforcing horizontal rural community connections [61], or forming a continuous, unified multi-level interlocking system of institutions and community services [62]. These perspectives also emphasize strengthening and building on existing systems to foster collaboration and coordination of community service resources as well as delivery agents, rather than causing interruption and fragmentation.

In the recently released *Design Standards for Rural Aging-in-Place Facilities (2023)*, the authors, who were part of the compilation team, have proposed two types of eldercare facilities in rural areas. The first type is the daycare center for the rural older population, which combines traditional daycare stations/centers with mutual aid centers. Based on the recognition of mutual assistance as an indispensable source of daily care for older rural people, it aims to provide daycare services such as recreation, meals, personal care, spiritual and cultural care, and opportunities for social gatherings. The second type is 24 h care for older villagers, which essentially extends the scope of application and functions of nursing homes/’Wubao’ institutes. This facility offers rehabilitation services for major illnesses and round-the-clock care, catering mainly to older individuals with difficulties or those in need of long and short stays. It also provides recreation, daycare, and accommodation options. The planning mainly relies on the existing eldercare and healthcare resources of the village. It involves integrating and upgrading these resources, capitalizing on the multi-functional nature of rural space, and strategically combining and updating facilities according to the actual conditions and unused houses. Notably, it proposes that the location of the rural eldercare facilities should be carefully chosen, leveraging the relative advantages of rural village committees and party committee locations with convenient transportation, well-established infrastructure, and natural aggregation of villagers. The primary criterion for site selection is to ensure proximity to these committees, prioritizing the convenience of villagers and maximizing resource utilization. This approach emphasizes capturing the favorable attributes of rural areas, including the spirit of kinship-based mutual help, abundant natural resources, rich traditional customs, and indigenous wisdom.

## 5. Conclusions

This paper, though historical data, existing literature, and reports from field study, presents the evolutionary process, motivations, characteristics of each historical stage, and progressive determinant relationship among the eldercare support patterns, public eldercare, and healthcare services, as well as the corresponding facilities for the senior group in China’s rural context, demonstrating the internal mechanisms and principles that drive the production of eldercare facilities in rural places. By acknowledging the social differences, the proposed framework in this paper allows the possibility of retrospection of the superstructure of the current status and problems of the existing facilities, leading to a deeper understanding of existing rural eldercare facilities. The aim is to glean valuable insights that can facilitate improved support for aging populations in rural China in the future.

China has witnessed significant transformations in its political and economic landscape since its establishment. Unlike Western societies with a relatively smooth and gradual evolution within a market-based economy, China’s social structure, encompassing ideology, traditions, forms of ownership, and reform trajectory, is uniquely intricate and unparalleled globally. Hence, the discourse surrounding its aging populace is also out of the ordinary [63]. The history of China’s rural development provides a comprehensive record of the ongoing reform process spanning 70 years. This scenario offers a distinctive prospect for comprehending the evolution of spatial material composition at diverse tiers, as impacted by the co-occurrence of two distinct forces: socialism and market economy. It is important to recognize that the rural–urban divide encompasses various dimensions, including policies related to land, income distribution, medical services, social security, grassroots organizational structures, and the specific characteristics and needs of older villagers. Therefore, it is imperative to treat rural aging as a distinct research focus, separate from the discussion of urban contexts that are often considered the default. This paper represents an initial endeavor to understand fundamentally the evolutionary trajectory of eldercare facilities as an architectural ‘outcome’ within a specific political and economic development context. However, certain limitations are apparent. Firstly, while a substantial amount of historical information and policy documents have been collected, some data are definitely bound to be out of reach, especially the precise number of eldercare facilities during specific periods, and only approximate figures and trends can be drawn. Secondly, rural China is currently undergoing tremendous and rapid transformations, and the understanding and research findings on eldercare services and facilities as part of this vast system may be impacted by both external and internal changes. Therefore, this research will require ongoing updates and sustained effort in the long term.

## Figures and Tables

**Table 1:** Stages of eldercare support patterns and services in rural China.

	Eldercare Support Patterns	Eldercare Services	Healthcare Services
**1949–1953**	**Self-organization before Collectivization**		
	Self-reliance, family care, and mutual assistance among the village	Not applicable; mutual assistance among older villagers	Fund-raising by villagers, agricultural cooperative, and doctors
**1954–1959**	**Social welfare fluctuated with Collectivism** Self-reliance and family care supported by the commune; minimal coverage and basic social welfare for ‘Wubao’ seniors	**The formation of the dual structure between urban and rural places under the People’s Commune System** Only basic support covering ‘Wubao’ seniors under the People’s Commune System	**Exploration and practice of Rural Cooperative Medical System** Parallel exploration of health service supply by the government and people themselves; the three-level Rural Cooperative Medical System based on People’s Commune; rapid increase in number of healthcare institutes in rural areas
**1960–1977**			**Fluctuation of RCMS and the domination of barefoot doctors** Self-sufficiency of healthcare services and medicine under the absence of Rural Cooperative Medical System; clinics and medical stations occupied by ‘barefoot doctors’
**1978–2005**	**Revival of social welfare after the reform of Household Contract Responsibility System** Self-reliance and family care supported by a limited pension security; minimal coverage and basic social welfare for ‘Wubao’ seniors	**The aggravating of dual structure under the Village Committee System** Very limited public services covering a wider range of people provided by institutions	**The trial and revival of RCMS** The revival of RCMS; establishment and wide coverage of New Rural Cooperative Medical System (NCMS)
**2006–2008**		**The breakdown of dual structure under the community system** Expanding the source of service; improving the equalization of public services between urban and rural areas	
**2009-**	**Multi-path exploration since the inception of building harmonious socialist society** Self-reliance and family care supported by an expanded pension security; developing social welfare covering a wider range of people supported by multiple subjects		**Systematization and equalization of public healthcare service** Merging healthcare services between urban and rural areas; combining of eldercare and healthcare

## Data Availability

The policy-related information in this paper is sourced from archived public policy documents of the Central People’s Government of the People’s Republic of China. These documents are accessible through the “Information Disclosure” of the official website: https://www.gov.cn/zhengce/xxgk/. Additional details about the fieldwork data in this paper can be found in the authors’ other published articles and by contacting the corresponding author directly.
